# Chondrosarcoma of the spine: A rare case with unusual presentation

**DOI:** 10.1186/1746-1596-1-39

**Published:** 2006-10-30

**Authors:** John Panelos, Spyridon Voulgaris, Evangelos Michos, Michael Doukas, Konstantinos Charalabopoulos, Anna Batistatou

**Affiliations:** 1 Department of Pathology University of Ioannina Medical School, Ioannina, Greece; 2 Department of Neurosurgery, University of Ioannina Medical School, Ioannina, Greece; 3 Department of Physiology, Clinical Unit, University of Ioannina Medical School, Ioannina, Greece

## Abstract

Chondrosarcoma is the third most common primary malignancy of bone, affecting primarily the pelvic and shoulder girdles and being extremely rare in the spine. Herein, we present a case of a 65-year-old woman with a rare chondrosarcoma of the spine, who presented with clinical symptoms from the lung metastasis. The neoplasm was grade II and exhibited overexpression of the p53 tumor suppressor protein. The latter has been associated with a high propensity for distant metastases.

## Background

Chondrosarcoma is a malignant tumor composed entirely of hyaline cartilage matrix and chondrocytes [6,12]. It is the third most common primary malignant neoplasm of bone after myeloma and osteosarcoma. The majority of patients are older than 50 years, with a peak of incidence in the fifth to seventh decade of life. More than two thirds of cases involve the bones of the pelvis, the shoulder and the proximal ends of femur and humerus [12]. Chondrosarcoma is extremely rare in the spine [1,10,11].

## Case Presentation

A 65-year-old woman, non smoker, visited our hospital with a 6-month history of persistent dry cough. Radiographic examination revealed the presence of a solitary nodule of the lung (right upper field), with morphologic features more consistent with metastasis. The patient mentioned a persistent, mild lumbar pain for the last two years and she had received nonsteroidal anti-inflammatory drugs. Upon a detailed physical examination a barely palpable mass at the lumbar area was noted. The reflexes of the lower extremities were normal. Laboratory examination showed no abnormalities, and all tumor markers had normal values. Magnetic resonance imaging (MRI) and CT-scan revealed an ill-defined osteolytic lesion of the L 5 vertebra with a splotch calcification, cortical permeation and extension to the surrounding soft tissues. In chest CT-scan the lung nodule was of similar consistency. An attempt to biopsy the lung nodule was made, but the material obtained was insufficient for diagnosis.

The patient underwent surgery in the spine, due to the danger of collapse of the L5 vertebra and the mass was exsiced.

**Figure 1:**
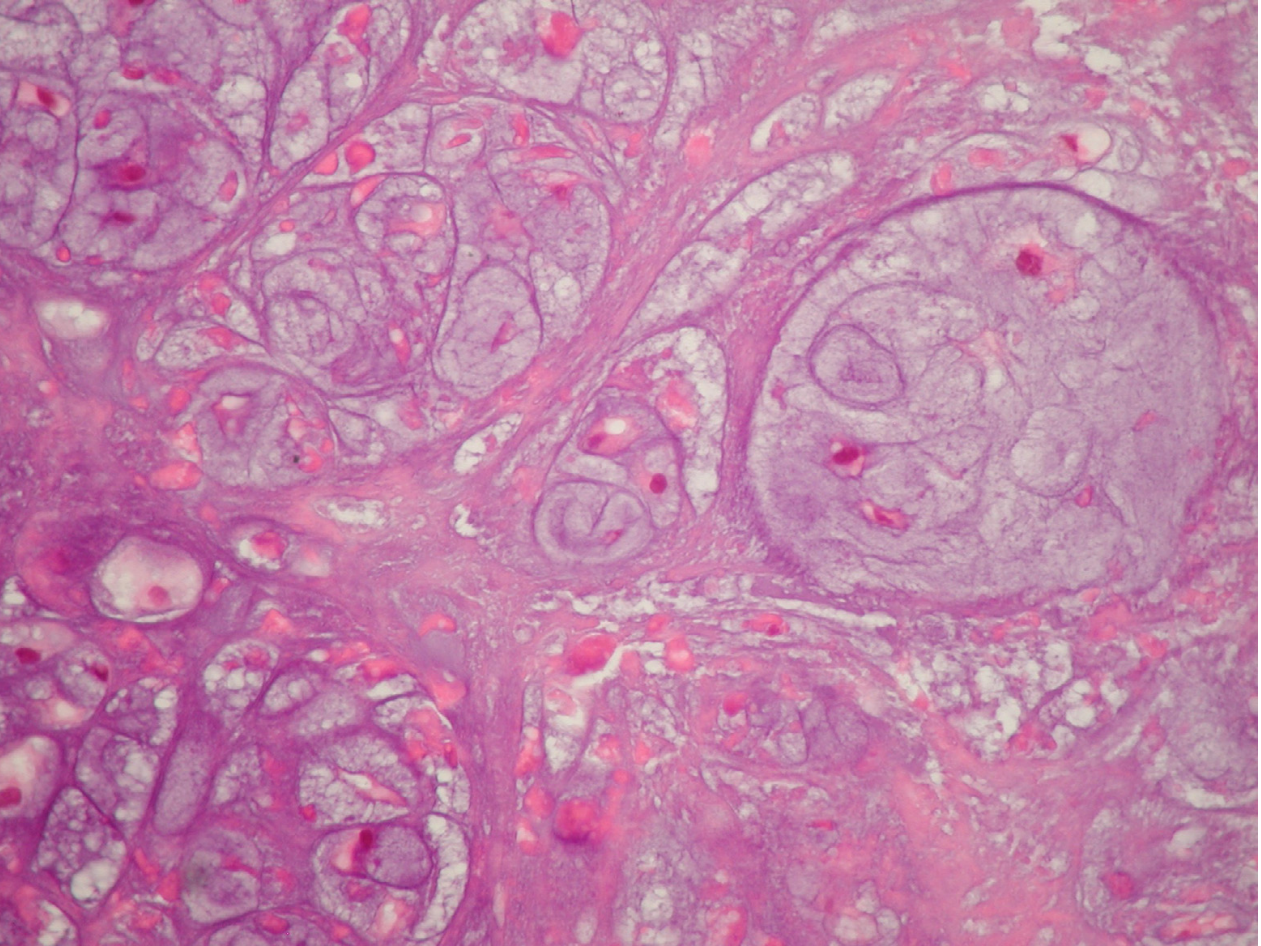
Multilobular neoplasm with chondroid matrix and nuclear pleiomorphism (haematoxylin and eosin X400).

**Figure 2:**
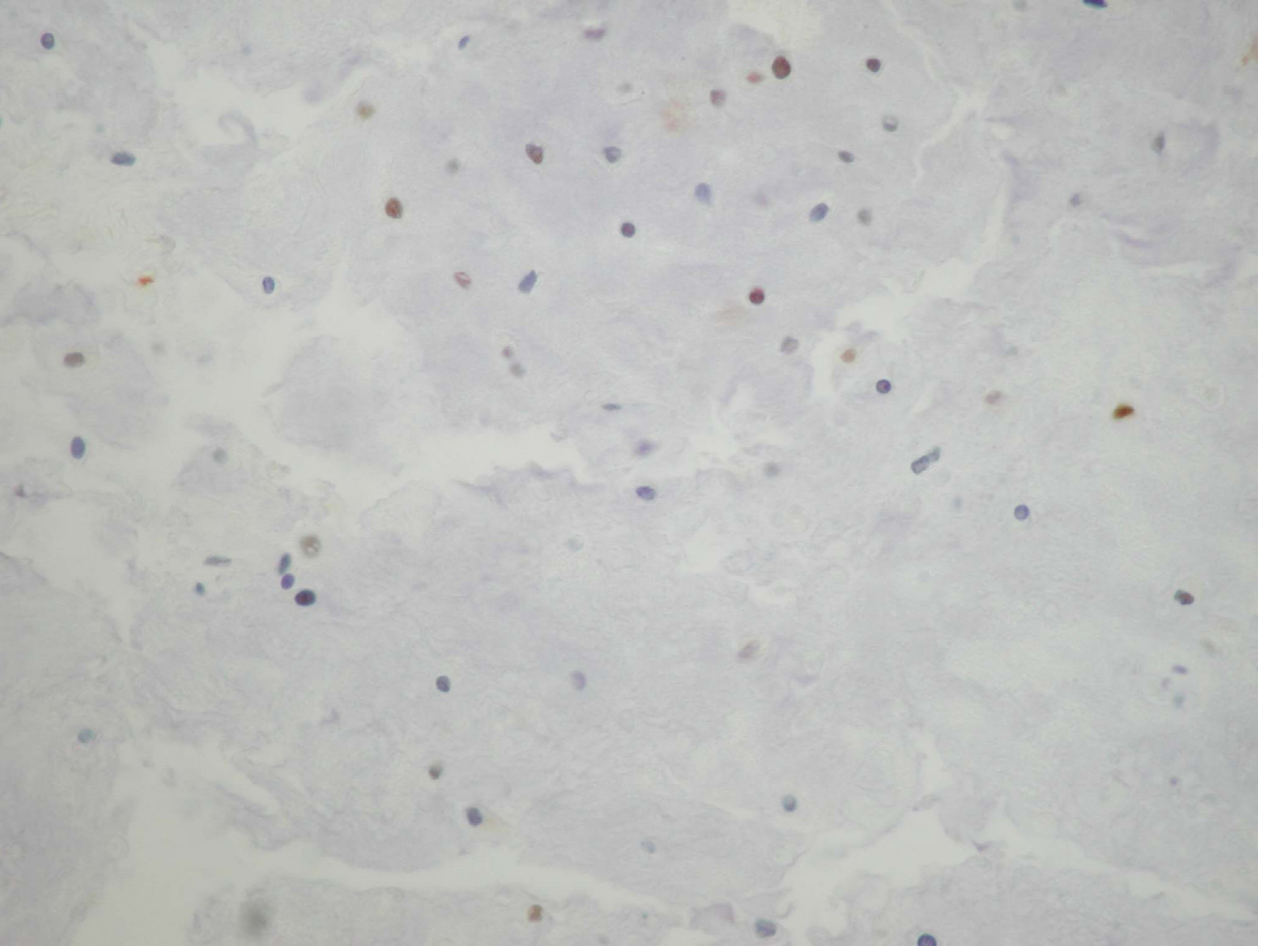
Increased nuclear immunoreactivity for p53 (DABX400).

**Figure 3:**
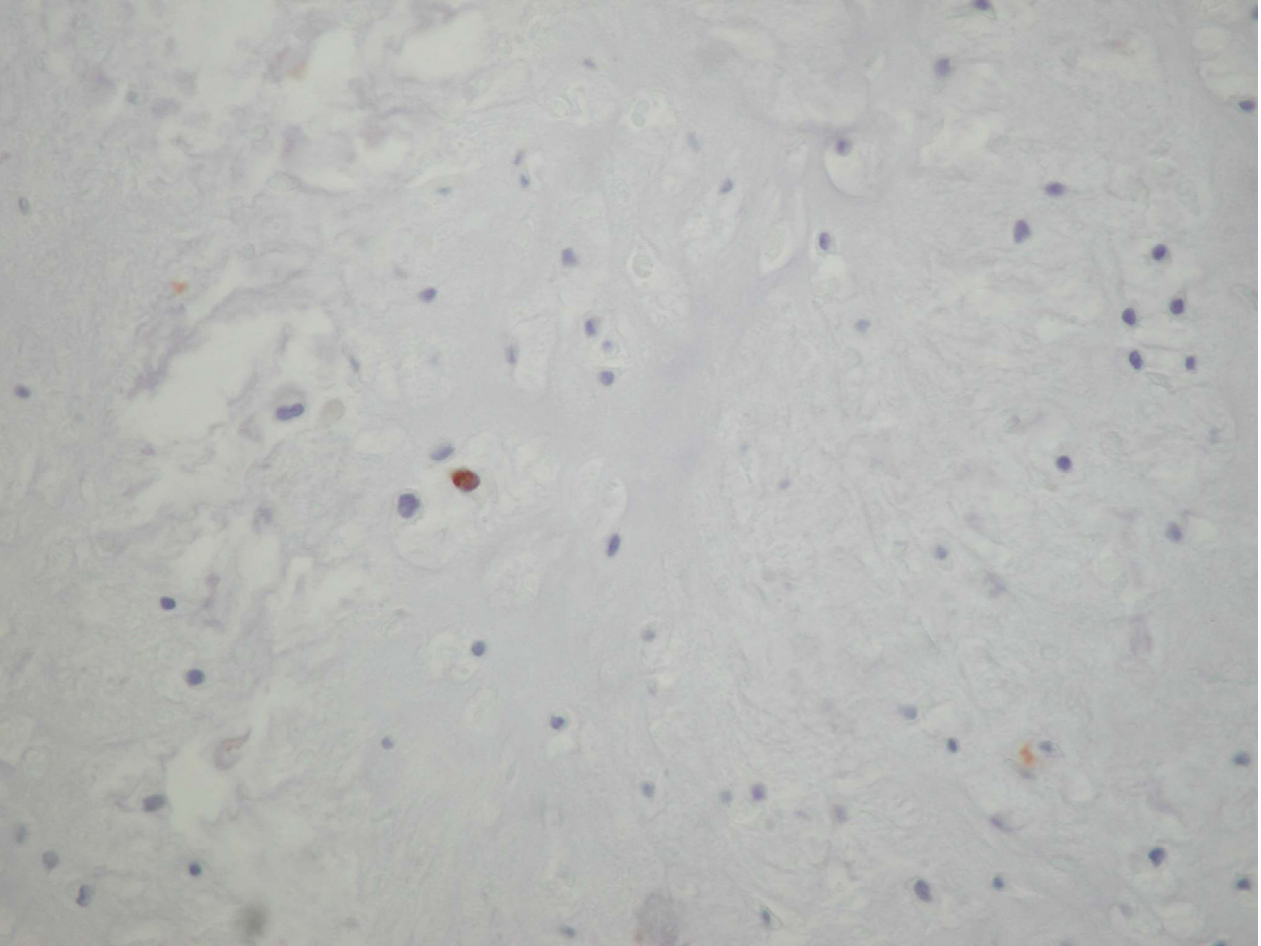
Rare nuclear immunoreactivity for MIB-1 (ki-67) (DABX400).

The histopathological examination revealed a moderately cellular multilobular neoplasm with chondroid and myxoid matrix and focal necrosis. Under high power view, multicellular lacunae of chondrocytes with moderate nuclear pleomorphism and hypercromasia were observed (Fig. 1). Mitotic figures were numerous. The tumor was diagnosed as chondrosarcoma, grade II. On immunohistochemical examination > 25 % of the neoplastic cells exhibited nuclear immunoreactivity for the tumor suppressor protein p53 (Fig. 2). Furthermore < 1 % of the neoplastic cells were in cell cycle, as estimated with the immunohistochemical marker MIB-1 (ki-67) (Fig. 3).

The patient underwent radiotherapy and is alive one year later.

## Discussion

The incidence of chondrosarcoma of the spine is reported to be 4–10% of all chondrosarcomas and 12% of all malignant neoplasms of the spine [1,10,11]. The main symptom is pain and sensory or motor deficit. The diagnosis is difficult, particularly from needle biopsy material, therefore open biopsy is recommended. The microscopic features of chondrosarcoma of the spine are similar with those of the most common appendicular tumors.

Chondrosarcomas are locally aggressive tumors with limited potential for metastasis. When metastases occur, they generally involve the lungs and appear late in the course of the disease. Wide surgical resection is the treatment of choice. Chondrosarcomas are divided into three grades according to the nuclear size, nuclear staining (hypercromasia) and cellularity [4]. Many studies have shown the significance of grading of chondrosarcoma in predicting the tumor behaviour and prognosis. Indeed, histological grade is the most important single predictor of local recurrence and metastasis.

Cytogenetic analyses have demonstrated a strong correlation between chromosomal abnormalities and histologic grade. Of the proto-oncogenes only the c-met gene which encodes for a transmembrane tyrosine kinase has been shown to be associated with the development of cartilaginous tumors [13]. Of the tumor suppressor genes, several studies have shown that altered patterns of p53 expression and TP53 mutations are detected primarily in high-grade, advanced tumors. The TP53 gene appears to be inactivated late in the course of chondrosarcoma and may be associated with disease progression. [2,3,7,12]. Altered p53 status has been correlated with high propensity for local recurrence and distant metastases even in grade I and grade II chondrosarcomas, indicating that it may represent a predictor of aggressive behavior that is independent of grade [8]. Furthermore, in a recent study, overexpression of p53 and Ki-67 in high-grade chondrosarcomas has been correlated with an unfavorable outcome [5]. The development and progression of malignant neoplasms has been associated with the production of different molecules, such as growth factors, that can be identified as biological markers as well. In chondrosarcomas, besides p53 and Ki-67, the expression of growth factors, i.e. TGFβ and PDGF and of VEGF has been associated with more aggressive behavior [5].

This is the first report on the p53 expression in a spinal chondrosarcoma. It is interesting that in our case, which presented initially with symptomatology from the lung metastasis, we noted p53 immunostaining in a high percentage of neoplastic cells. Thus, it appears that in spinal chondrosarcomas, similar to the appendiceal ones, the metastatic potential may be correlated with altered p53 status. Further studies, with a series of spinal chondrosarcomas and possibly with the use of tissue microarrays [9] are needed for definite conclusions.

## Competing interests

The author(s) declare that they have no competing interests.

## Authors' contributions

JP, KC and AB have been involved in conceiving and drafting the manuscript. JP and MD have been involved in the literature search. EM and SV have worked-up and treated the patient. All authors read and approved the final manuscript.
